# Myxedema Coma as a Presentation of Panhypopituitarism Secondary to Traumatic Brain Injury

**DOI:** 10.1155/2024/3588840

**Published:** 2024-10-16

**Authors:** Diego Rivas-Otero, Tomás González-Vidal, Pedro Pujante Alarcón, Elías Delgado Álvarez, Edelmiro Menéndez Torre

**Affiliations:** ^1^Department of Endocrinology and Nutrition, Central University Hospital of Asturias/University of Oviedo, Oviedo, Spain; ^2^Endocrinology, Nutrition, Diabetes and Obesity (ENDO) Group, Health Research Institute of Asturias, Oviedo, Spain; ^3^Department of Medicine, University of Oviedo, Oviedo, Spain; ^4^Centre for Biomedical Network Research on Rare Diseases (CIBERER), Carlos III Health Institute, Madrid, Spain

**Keywords:** hypopituitarism, myxedema coma, traumatic brain injury

## Abstract

**Background/Objective:** Myxedema coma typically presents with decreased level of consciousness and hypothermia, often due to thyroid pathology. In central causes, normal thyroid-stimulating hormone (TSH) levels may delay diagnosis. The purpose of this report is to describe a patient with a history of head trauma who presented with myxedema coma as a manifestation of panhypopituitarism.

**Case Report:** The admitted patient was a 52-year-old man who presented with mental and physical slowness, drowsiness, and weakness. He also had hypotension, hypoglycemia, and low oxygen saturation. Initial evaluation revealed severe pericardial and bilateral pleural effusions, plasma TSH of 2.42 mU/L (normal range 0.25–5.00 mU/L), and plasma adrenocorticotropic hormone (ACTH) of 7.1 pg/mL (normal range 5.2–40.3 pg/mL). Later, his condition deteriorated with anasarca and coma. Signs of improvement were noted after intravenous corticosteroid administration. A subsequent blood test was conducted, which showed a free thyroxine (FT4) level of 0.14 ng/dL (normal range 0.93–1.70 ng/dL). A cranial magnetic resonance scan revealed posttraumatic lesions. The patient's family later admitted head injuries at home. Treatment with intravenous levothyroxine was initiated, resulting in improvement and subsequent discharge in perfect alertness.

**Conclusion:** Hypopituitarism should be suspected in patients with head trauma and symptoms of hormone deficiency. Advanced clinical forms, such as myxedema coma, may also occur. Pituitary hormone levels might be in the normal range, so target gland hormones should be assessed to reach a diagnosis. In the case of suspected central hypothyroidism, requesting only TSH levels may result in a missed diagnosis. For this reason, both TSH and FT4 levels should be measured when central hypothyroidism is suspected.

## 1. Introduction

Myxedema coma is a clinical condition resulting from severe and long-term thyroid hormone deficiency, which overwhelms the compensatory mechanisms of the organism. The incidence is low, around 0.22 cases per million population in Western countries, with a female predominance (80%) as hypothyroidism is more common in women, and a mortality rate that ranges from 20% to 60% [1]. The level of consciousness in patients with myxedema coma is generally impaired and may range from drowsiness to coma. A recent retrospective study showed that 98% of patients with myxedema coma had an altered level of consciousness (Glasgow Coma Scale [GCS] ≤ 14), and 52% were in coma (GCS < 9) [2]. The second most common clinical feature is hypothermia, present in 40%–100% of cases [3]. Patients with myxedema coma often present with dilutional hyponatremia [4], which can contribute to focal or generalized seizures in up to 25% of these patients [5]. In a previous study, 57% of patients had hemodynamic instability (sequential organ failure assessment [SOFA] ≥ 2), 50% were bradycardic, and 18% had hypoglycemia [2]. Pericardial [6] and pleural effusions [7], which are predominantly exudative [6], may be present.

Around 93% of the patients with myxedema coma have a primary etiology (i.e., thyroid pathology) [2], so secondary or tertiary causes (i.e., pituitary or hypothalamic pathology) such as traumatic brain injury (TBI) are uncommon. TBI has been associated not only with thyroid axis impairment but also with potential injury to any pituitary axis in 20% to 80% of patients [8, 9]. However, there is still a lack of awareness of this association. We present the case of a patient with panhypopituitarism after TBI, who presented with myxedema coma.

## 2. Case Report

The patient was a 52-year-old male cleaner who smoked 20 cigarettes a day and consumed 1 L of wine per day. He had a history of obesity (body mass index of 32 kg/m^2^), arterial hypertension, dyslipidemia, and mild hepatic fibrosis. He had no history of pituitary disorders. He was taking 1 mg of pitavastatin daily as a chronic medication.

The patient presented with a 4-month insidious history of progressive mental and physical slowing, drowsiness, and weakness. In addition, he presented with cough and dyspnea of 24-h duration, which prompted his family to take him to the emergency room (ER). Initial assessment revealed hypotension (blood pressure of 80/50 mmHg), hypoglycemia (capillary blood glucose of 51 mg/dL), an oxygen saturation by pulse oximeter of 92%, and a normal body temperature (36.3°C). Heart sounds were diminished on auscultation, and crackles were heard at the base of the lungs. A blood test was then ordered, which showed mild acidosis (pH 7.33) with a lactate of 3.3 mmol/L, neutrophilia (neutrophil count of 10.62· 10^3^/µL, normal range 2.00–7.00· 10³/µL), hyponatremia (plasma sodium of 123 mmol/L), and acute kidney injury (glomerular filtration rate of 38 mL/min/1.73 m^2^, previous determination was >90 mL/min/1.73 m^2^). Subsequently, a chest X-ray revealed cardiomegaly and bilateral pleural effusions, prompting a chest computed tomography (CT) scan, which showed severe pericardial effusion and bilateral pleural effusions. Consequently, the patient was admitted to the intensive care unit (ICU) 24 h after arrival at the ER.

A blood test was performed 24 h after admission to the ICU (day 2 since arrival at the ER) and showed both normal thyroid-stimulating hormone (TSH) and adrenocorticotropic hormone (ACTH) (Table 1, Column 1). TSH was available on the same day of sampling, and ACTH was available 72 h after sampling. No other hormonal assays were conducted that day.

During the first hours in the ICU, nonpitting edema developed in the lower limbs, and the patient fell into a state of stupor. His new clinical conditions progressed rapidly to anasarca and coma, respectively, and hypotension was persistent with the need for high doses of vasopressors. The patient underwent evaluations by psychiatry, neurology, and internal medicine, with no findings in multiple complementary tests (cultures, hemodynamic and autoimmune studies, deposition disease studies, serologies, body imaging tests, and cranial CT scan). The pericardial and pleural effusions were drained and analyzed, revealing noninfectious exudate.

Given the clinical deterioration and refractory arterial hypotension, intravenous corticosteroid therapy was started in the ICU 3 days after the admission, at a dose of 100 mg intravenous hydrocortisone every 8 h. The patient's clinical condition gradually improved after initiation of corticosteroid treatment, emerging from the coma but remaining obtunded or sometimes stuporous. Hypotension and anasarca persisted. Morning plasma cortisol levels were measured after initiation of intravenous hydrocortisone, resulting in falsely elevated levels (Table 1, Column 2). No further hormone determinations were made on the following days. After 12 days on intravenous corticosteroids, they were discontinued, and oral hydrocortisone was started at a dose of 20 mg every 6 h. At that point, the ICU physicians requested an endocrinology assessment. A comprehensive hormonal profile was requested after the endocrinology evaluation, with some initial results available in less than 24 h (Table 1, Column 3). Plasma cortisol levels were measured while the patient was being treated with hydrocortisone, so they were falsely elevated again (Table 1, Column 3). Plasma levels of free thyroxine (FT4) and free triiodothyronine (FT3) were significantly decreased (Table 1, Column 3). These findings, together with normal TSH levels, led to the diagnosis of myxedema coma due to central hypothyroidism. The presence of this diagnosis, together with the patient's clinical improvement after previous corticosteroid administration, also raised a high suspicion of coexisting central adrenal insufficiency.

As a result of not measuring FT4 levels at the initial screening, the diagnosis of central hypothyroidism was delayed by 13 days from the first normal TSH measurement. On the same day FT4 levels were assessed, treatment was initiated with 500 µg of intravenous levothyroxine as a loading dose, followed by 100 µg/day starting the next day. After a total of 6 days on intravenous levothyroxine, he was switched to 125 µg of oral levothyroxine daily. A cranial magnetic resonance imaging (MRI) scan was requested. Upon further history taking with the family, they recalled several falls in the previous months related to alcohol intoxication. Those falls were not witnessed, and the patient did not present obvious loss of consciousness or external bleeding. The family also reported that the patient had shown marked cold intolerance in the previous summer months, persistent new-onset constipation, and weight gain.

**Figure 1:**
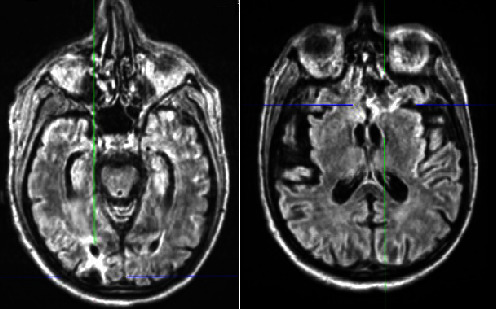
Cranial magnetic resonance imaging scan of the patient (axial, T1-weighted), which revealed left frontobasal and right occipital gliotic–malacic lesions with marginal hemosiderin deposits, suggesting posttraumatic or postischemic sequelae.

**Figure 2:**
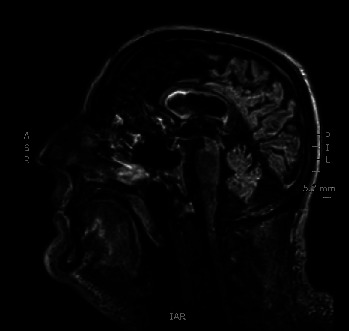
Normal pituitary gland of the patient. ASR, anterior-superior right; IAR, inferior-anterior right; PIL, posterior-inferior left.

At 48–72 h, we received the remaining results of the blood test requested by endocrinology after the first evaluation, updating the diagnosis to “panhypopituitarism” after documenting hypogonadotropic hypogonadism and both insulin-like growth factor 1 (IGF-I) and prolactin deficiencies (Table 1, Column 3). At this point, it had been 17 days since his arrival at the hospital. The patient continued to improve, and 22 days after his arrival at the hospital, he was transferred from the ICU to the endocrinology ward. Two days later, we obtained MRI images, which revealed posttraumatic and/or postischemic left frontobasal and right occipital gliotic–malacic lesions with marginal hemosiderin deposits (Figure 1), without signs of acute/subacute pathology and with a normal pituitary gland (Figure 2). Considering the clinical presentation, the reported history, the localization of the cranial lesions, and the patient's response to treatment, we diagnosed panhypopituitarism resulting from TBI.

The patient's condition improved over the following days, regaining alertness, with progressive resolution of edema and tolerating oral treatment. Twenty-nine days after arriving at the ER, he was eventually discharged on oral hydrocortisone (20 mg in the morning and 10 mg in the afternoon) and levothyroxine (125 µg/day, 1.47 µg/kg), choosing not to treat the remaining hormonal deficiencies. Four weeks after discharge, he had normal thyroid hormone levels (Table 1, Column 4). We requested a comprehensive plasma hormone profile 2 months after hospital discharge. At that time, the somatotropic and gonadal axis deficiencies had spontaneously normalized, with persistence of the corticotropic axis deficiency (Table 1, Column 5). These plasma cortisol levels were measured after 24 h without oral hydrocortisone, as the patient was asked not to take the afternoon dose of hydrocortisone the day before sampling. Thyroid hormones remained normal while maintaining the same dose of levothyroxine prescribed at discharge (Table 1, Column 5).

## 3. Discussion

TBI causes pituitary hormone deficiency more often than thought. Previous studies in patients with TBI showed a frequency of hypopituarism of 20%–80% [8, 9]. Hormone deficiency after TBI can take years to be diagnosed. A study that reviewed 367 patients with TBI-induced hypopituitarism revealed that 15.3% of patients were diagnosed 5 years after the TBI, while 6.0% of patients were diagnosed 20 years after the TBI [10]. All pituitary axes can be impaired (i.e., hormone deficiencies) in the short- and long-term in patients with TBI [8, 9, 11]. According to these studies, the frequency of pituitary axis impairment, from the highest to lowest, is somatotrophic axis, gonadal axis, corticotropic axis, and thyroid axis [8, 10–12]. Thyroid and gonadal axis deficiencies can be diagnosed based on basal hormone levels [13]. However, to diagnose somatotropic and corticotropic axis deficiencies, dynamic stimulation testing is required [13]. It should be noted that in emergency and intensive care conditions, a basal cortisol level of ≤11 µg/dL in the acute phase of head trauma suggests ACTH deficiency [13].

The causes of hypopituitarism after TBI appear to be diverse. Due to its anatomical location, the pituitary gland is particularly susceptible to direct damage after TBI, which can result in vascular damage and subsequent hormone deficiency [13]. In patients with fatal TBI in which autopsies were performed, over 70% have some degree of pituitary infarction, and up to 40% have hypothalamic microhemorrhages [14]. The location of the hormone-producing cells in the pituitary gland may also be significant. The ventromedial portion of the pituitary contains cells that produce both ACTH and TSH. These cells receive greater blood flow than those that produce growth hormone (GH), follicle-stimulating hormone (FSH), and luteinizing hormone (LH), which are located in the periphery [15]. This could explain why somatotropic and gonadal axis deficiencies are more prevalent after TBI. After the direct injury, a number of secondary injuries to the pituitary gland (e.g., impaired regulation of cerebral blood flow, increased intracranial pressure, and edema formation around the pituitary gland) may either perpetuate or exacerbate the initial injury, resulting in chronic hormone deficiency [13]. Genetic and autoimmune mechanisms may also play a role in the development of chronic pituitary dysfunction after TBI [13]. It is unclear whether a greater magnitude of the TBI is associated with a higher prevalence of hypopituitarism. Some studies support this association [16, 17], while others do not [8, 14]. There does not appear to be a link between epidural hemorrhage, subdural hematoma or intracranial hemorrhage, and hormone deficiency [17]. However, neurosurgery is a clear independent risk factor for hypopituitarism [17]. Subarachnoid hemorrhage, diffuse axonal injury, skull base fractures, or older age may also be risk factors [8, 9]. Continuous slight bleeding into the subarachnoid space can lead to brain hemosiderin deposition [18]. Since the cause of hemosiderin deposition in our patient was unknown, it is conceivable that these hemosiderin deposits could be a sequela of undetected intracranial bleeding. Furthermore, alcohol consumption is a significant risk factor for both TBI [19] and the development of functional hypopituitarism independent of TBI [20]. Chronic alcohol consumption, as in the case of our patient, has been linked with reduced plasma levels of ACTH, TSH, GH, and LH [20]. Similarly, chronic nicotine consumption, also present in our patient, has been associated with low plasma levels of TSH, prolactin, and IGF-I [21, 22].

Recovery of damaged pituitary axes after TBI is variable. In a prospective study of 70 patients with TBI, all patients who had panhypopituitarism 3 months after the trauma still had it at 12 months [8]. However, the same study showed that isolated deficiencies at 3 months are more likely to recover at 12 months [8]. We must also consider that the development of new hormone deficiencies has been described in some patients during follow-up, despite an initial normal screening [17]. Our patient's gonadal and somatotropic axes recovered, but corticotropic and thyrotropic did not. The recovery of the gonadal axis has been reported in 33%–50% of patients, and some authors suggest that damage to this axis may have a functional origin in the context of acute stress [8, 13]. Recovery from severe GH deficiency has also been reported [8]. Recovery rates for the corticosteroid axis range from 16% to 70% [8, 11], while recovery rates for the thyroid axis range from 0% to 50% [8, 11]. The patient in the present case report had long-term impairment of the thyroid and corticotropic axes, two axes that are rarely affected after TBI [8, 10–12, 17] but which may not recover and thus become chronic deficiencies [8, 11].

The diagnosis of myxedema coma requires a high index of suspicion based on the patient's clinical presentation and is confirmed by low blood levels of FT4 and FT3. Diagnostic scales have been proposed [23], but their use in clinical practice is not widespread. Treatment should be given in an ICU as these patients may present with multiple cardiopulmonary, neurological, or electrolyte decompensations. Severe hypothyroidism can interfere with adequate ACTH secretion, resulting in subnormal cortisol response to stress [24]. Therefore, corticosteroid therapy (e.g., 100 mg intravenous hydrocortisone every 8 h) is recommended for myxedema coma, especially when refractory shock is present and until adrenal insufficiency has been ruled out. The latter is particularly relevant because treatment of severe hypothyroidism without prior treatment of adrenal insufficiency can precipitate an adrenal crisis as a side effect of high doses of levothyroxine [25]. Treatment with thyroxine (T4) monotherapy is then recommended, starting with an intravenous loading dose of 200–600 µg [25, 26] and continuing with 100 µg daily until the patient can tolerate oral administration. The role of triiodothyronine (T3) treatment is controversial as it may induce arrhythmias or ischemia: some guidelines recommend it only in selected patients, such as young patients with no history of heart disease who have failed to show clinical improvement when treated with T4 [25], while others suggest combined treatment with T4 and T3 from the outset [26].

In conclusion, when suspecting a hormone deficiency, it is important to consider that the cause might be secondary or tertiary. In such cases, pituitary hormone levels may be low or inappropriately normal, which means that a normal pituitary hormone determination (e.g., normal TSH levels) does not rule out a hormone deficiency, and a study of the target gland hormones (e.g., FT4 and FT3) should be requested. TBI often results in central hormone deficiencies, so hypopituitarism should be suspected in patients with TBI who present symptoms consistent with hormone deficiency. Some of these deficiencies are detected late, and occasionally they may present in advanced clinical forms, such as the myxedema coma seen in our patient. Severe thyroid hormone deficiency should be suspected in patients who present with decreased level of consciousness plus hypothermia, hyponatremia, hypoglycemia, hypotension, and/or bradycardia. If the entity is confirmed, treatment should be initiated promptly with both glucocorticoids and thyroid hormone.

## Figures and Tables

**Table 1:** Monitoring patient's plasma hormone profiles and date of sampling in relation to his arrival at the emergency room.

Plasma hormones	Hospitalized			Outpatient		Reference range	Units
	Day 2	Day 3^a^	Day 14^a,b^	Day 58	Day 90		
TSH^c^	2.4	—	2.53	1.60	0.98	0.25–5.00	mU/L
Free thyroxine (FT4)^c^	—	—	0.14	1.40	1.28	0.93–1.70	ng/dL
Free triiodothyronine (FT3)^c^	—	—	0.39	2.26	—	2.00–4.00	pg/mL
Cortisol^c^	—	86.60	32.60	—	0.60	6.24–18.00	µg/dL
ACTH^d^	7.1	—	10.4	—	—	5.2–40.3	pg/mL
FSH^d^	—	—	3.2	—	6.5	1.5–12.4	U/L
LH^d^	—	—	3.2	—	5.4	1.7–8.6	U/L
Insulin-like growth factor 1 (IGF-I)^d^	—	—	35	—	127	48–209	ng/mL
Prolactin^d^	—	—	2.00	—	5.33	4.04–15.20	ng/mL
Total testosterone^d^	—	—	1.35	—	3.59	1.93–7.40	ng/mL
Bioavailable testosterone^d^	—	—	2.01	—	6.82	3.59–11.00	nmol/L
Sex hormone-binding globulin (SHBG)^d^	—	—	33.2	—	24.6	19.3–76.4	nmol/L

Abbreviations: ACTH, adrenocorticotropic hormone; FSH, follicle-stimulating hormone; LH, luteinizing hormone; TSH, thyroid-stimulating hormone.

^a^Consider that the patient was on corticosteroid treatment at that time.

^b^Treatment with levothyroxine was initiated after evaluation of the results of this analysis and was continued throughout the follow-up period.

^c^Available ≤24 h after sampling.

^d^Available in 48–72 h after sampling.

## Data Availability

All data underlying the case report are available as part of the article, and no additional source data are required.
